# Integrative genomic analysis identified common regulatory networks underlying the correlation between coronary artery disease and plasma lipid levels

**DOI:** 10.1186/s12872-019-01271-9

**Published:** 2019-12-23

**Authors:** Liuying Chen, Yinghao Yao, Chaolun Jin, Shen Wu, Qiang Liu, Jingjing Li, Yunlong Ma, Yizhou Xu, Yigang Zhong

**Affiliations:** 1 Zhejiang Chinese Medical University, Hangzhou, China; 2 State Key Laboratory for Diagnosis and Treatment of Infectious Diseases, The First Affiliated Hospital, Collaborative Innovation Center for Diagnosis and Treatment of Infectious Diseases, Zhejiang University School of Medicine, Hangzhou, China; 3 Nanjing Medical University, Nanjing, China; 4 Department of Cardiology, Affiliated Hangzhou First People’s Hospital, Zhejiang University School of Medicine, Hangzhou, China

**Keywords:** Coronary artery disease, Lipid levels, Multi-omics, Susceptible genes, Pathways, GWAS

## Abstract

**Background:**

Coronary artery disease (CAD) and plasma lipid levels are highly correlated, indicating the presence of common pathways between them. Nevertheless, the molecular pathways underlying the pathogenic comorbidities for both traits remain poorly studied. We sought to identify common pathways and key driver genes by performing a comprehensive integrative analysis based on multi-omic datasets.

**Methods:**

By performing a pathway-based analysis of GWAS summary data, we identified that lipoprotein metabolism process-related pathways were significantly associated with CAD risk. Based on LD score regression analysis of CAD-related SNPs, significant heritability enrichments were observed in the cardiovascular and digestive system, as well as in liver and gastrointestinal tissues, which are the main regulators for lipid level.

**Results:**

We found there existed significant genetic correlation between CAD and other lipid metabolism related traits (the smallest *P* value < 1 × 10^− 16^). A total of 13 genes (e.g., *LPA*, *APOC1*, *APOE* and *SLC22A3*) was found to be overlapped between CAD and plasma lipid levels. By using the data-driven approach that integrated transcriptome information, we discovered co-expression modules associated prominently with both CAD and plasma lipids. With the detailed topology information on gene-gene regulatory relationship, we illustrated that the identified hub genes played important roles in the pathogenesis of CAD and plasma lipid turbulence.

**Conclusion:**

Together, we identified the shared molecular mechanisms underlying the correlation between CAD and plasma lipid levels.

## Background

Coronary artery disease (CAD) is one of the leading causes of death globally [1]. Plasma lipid levels, including low-density lipoprotein (LDL) cholesterol, high-density lipoprotein (HDL) cholesterol, triglycerides and total cholesterol, are all associated with regulation of the risk for CAD. Further, The INTERHEART study indicated that 45% of heart attacks in Western Europe are due to abnormal blood lipid levels [2].

CAD and blood lipid levels are both heritable, with the genetic contribution estimated to be 40–60%. Genome-wide association studies (GWAS) have successfully identified more than hundreds of risk loci for CAD and plasma lipid levels [3–6]. Importantly, several genetic studies also suggest the existence of shared polygenic pleiotropy between CAD and blood lipids [4, 7, 8]. For example, the genes *APOA5*, *TRIB1* and *APOC3*, which were significantly associated with plasma lipids, also showed prominent risk to CAD [9, 10]. However, due to lack of multi-dimensional data integration analysis, the underlying mechanistic insights into the pathogenesis of comorbidity remain largely unknown.

In recent years, large-scale genetic association studies have yielded new insights into the genetic architecture of CAD and blood lipid levels, which enabled us to investigate the genetic etiology of comorbidity. As the fact that pleiotropy is pervasive, many relevant diseases or traits are commonly associated with the same underlying causal variants. The emerging challenge in today research is how to interpret the functional effects of common genetic signals between diseases and their risk factors. In addition, the cell-type-specific regulatory elements that control specific cell functions also increase the difficulties to identify key disease pathways and processes.

In the current investigation, we conducted a systematic analysis with the goal of revealing the underlying genetic architecture of CAD and shared gene regulatory network with plasma lipids using large-scale GWAS summary and gene expression data. By performing a transcriptome analysis in three relevant tissues, we modeled co-expression networks and identified common trait-associated modules shared between CAD and plasma lipids. Finally, we integrated topological gene regulatory networks to identify hub connected genes for both traits.

## Methods

### GWAS summary data sets

#### Dataset #1 for coronary artery disease

We obtained summary statistics from a large GWAS meta-analysis comprising more than 120,000 cases and 339,115 controls (Additional file 2: Table S1) [11]. Complete GWAS summary statistics were downloaded from the CARDIoGRAMplusC4D Consortium website (http://www.cardiogramplusc4d.org/data-downloads/).

#### Dataset #2 for plasma lipid levels

We obtained a published GWAS meta-analysis association data for lipid levels from Center for Statistical Genetics. This study was a joint analysis that examined 188,577 individuals that were genotyped with two platforms from multiple studies [12]. Complete GWAS summary statistics were downloaded from the website (http://csg.sph.umich.edu/willer/public/lipids2013/).

### Transcriptome data sets

There were three gene expression datasets obtained from Gene Expression Omnibus (GEO) database (Additional file 2: Table S2). For GSE30169, we filtered samples treated with 40 μg/ml oxidized 1-palmitoyl-2-arachidonoyl-sn-glycero-3-phosphatidylcholine (Ox-PAPC), which left 307 remaining normal primary human aortic endothelial cells. For GSE7965, adipose tissue samples from 701 individuals were included in the analysis. For GSE24335, 651 samples with expression profile of liver tissue were included in the analysis.

### Gene set analysis by GWAS summary statistics

We used the Multi-marker Analysis of GenoMic Annotation (MAGMA) [13] to test for enrichment of well-documented gene-sets, including data sources from KEGG, GO, BioCarta [14], and Reactome [15], with CAD. The SNPs were assigned to all protein-coding genes (or within a region extended − 30 kb upstream and + 10 kb downstream of the gene) based on the autosome of NCBI 37.3 coordinates. After SNP annotation, there were 18,410 genes containing SNPs in genotype data. For the gene set analysis, we restricted the analysis to 4608 pathways comprising 5–300 genes. MAGMA’s built in empirical multiple testing corrections were used to correct raw *P* values with 10,000 permutations.

### Partitioning heritability for CAD loci by cell-type-specific annotation

The polygenic contributions for cell-type-specific functional elements were estimated by linkage disequilibrium (LD) score regression analysis [16]. For CAD summary data, only common SNPs (MAF > 1%) presented in the HapMap version 3 data set were included in analysis model. LD scores were calculated by the 1000 Genomes Project Phase 1 EUR reference panel. As described by Finucane et al. [16], we first created a “full baseline model” with a total number of 53 overlapping functional categories. For cell type specific analysis, we used annotations from ten groups, including adrenal/pancreas, central nervous system (CNS), cardiovascular, connective/bone, gastrointestinal, immune/hematopoietic, kidney, liver, skeletal muscle, and others.

### Tissue/cell-type expression enrichment analysis

DEPICT analysis (Data-Driven Expression-Prioritized Integration for Complex Traits) [17] was used to test for enrichment of tissues or cell types where CAD-related gene are highly expressed. Firstly, we used PLINK v1.07 [18] to identify independent SNPs with *P* value less than 1 × 10^− 5^ from CAD GWAS summary, LD information was provided by the 1000 Genomes Project Phase 1 EUR reference panel. Then, we took advantage of the build-in data sets from DEPICT consisting of 209 tissue/cell types assembled from 37,427 human microarray samples for expression enrichment analysis.

### Genetic correlation analysis

We used the LD score regression method [19, 20] to profile the pattern of genetic correlations between CAD and lipid metabolism related traits, including low-density lipoprotein (LDL) cholesterol, high-density lipoprotein (HDL) cholesterol, triglycerides, total cholesterol, BMI, and waist-hip ratio. Quality control steps were adopted from LD scores default procedures, including imputation quality > 0.9 and MAF > 0.1. Moreover, all SNPs retained to analysis were merged to SNPs in HapMap 3 reference panel.

### Building gene co-expression network modules

CAD and lipid metabolism related tissues (including liver, aortic endothelial cells and adipose) transcriptome data were obtained from GEO datasets (https://www.ncbi.nlm.nih.gov/gds/) (Additional file 2: Table S2). Low-expressed and non-varying genes in each dataset were filtered to avoid noise, results in an average of 12,000 genes to be included in the following analysis. We applied the Weighted Correlation Network Analysis (WGCNA) [21] to construct gene co-expression modules. A number of 30 were set for minimum module size. We chose 0.1 as cut line in the dendrogram to merge similar modules (corresponding to correlation of 0.9).

### Identification of co-expression modules with over-representation of genetic association signals

We conducted Marker Set Enrichment Analysis (MSEA) to identify genetically perturbed co-expression modules for each phenotype using the Mergeomics pipeline [22, 23]. For the current analysis, MSEA takes three elements into workflow: (1) summary data for each GWAS (CAD, HDL, LDL, TC, TG, BMI, and WHRadjBMI); (2) assignment of SNPs to their corresponding genes; (3) functionally related gene sets generated from co-expression module.

### Identification of hub genes using weighted key driver analysis (wKDA)

The Mergeomics pipeline offers a function to detect key drivers and hub genes using detailed topology information on gene regulatory relationships [23]. We used GIANT networks [24] from three tissues (aorta, adipose and liver), which provide detailed interactions between genes according to independent gene expression datasets and protein interaction information. All genes in the CAD-associated module that also showed nominal significance in lipid metabolism related traits (*P* < 0.1) were mapped into GIANT networks with edges information, which support tissue-specific function interactions.

## Results

### CAD associated pathways are enriched in lipoprotein metabolism processes

To reveal the genetic architecture of CAD, we first performed pathway analysis to test the associations of predefined functional gene-sets, including KEGG, GO, BioCarta, and Reactome (see Methods for details). 4608 pathways with a size 5–300 genes per pathway were retained for downstream analyses in consideration of appropriate specificity and high efficiency. After corrections for multiple testing by permutation tests, 12 significant enriched pathways with corrected *P* value < 0.05 were identified (Additional file 2: Table S3). The top ranked pathway was collagen type IV (*P* = 1.32 × 10^− 09^), consisting of 6 genes coding type IV collagen proteins. Notably, two-thirds of pathways reached significant associations involved in lipoprotein metabolism and cholesterol and triglyceride homeostasis. These pathways contained 10 common genes, i.e., *LDLR*, *LPA*, *PLG*, *APOE*, *LIPA*, *LPL*, *APOB*, *ABCG8*, *ABCG5*, *APOC4* (Additional file 2: Table S4), that were significantly associated with CAD (*P* < 2.72 × 10^− 06^) by using the MAGMA analysis.

### CAD related SNPs/genes were functionally annotated at liver and gastrointestinal tissues

**Fig. 1:**
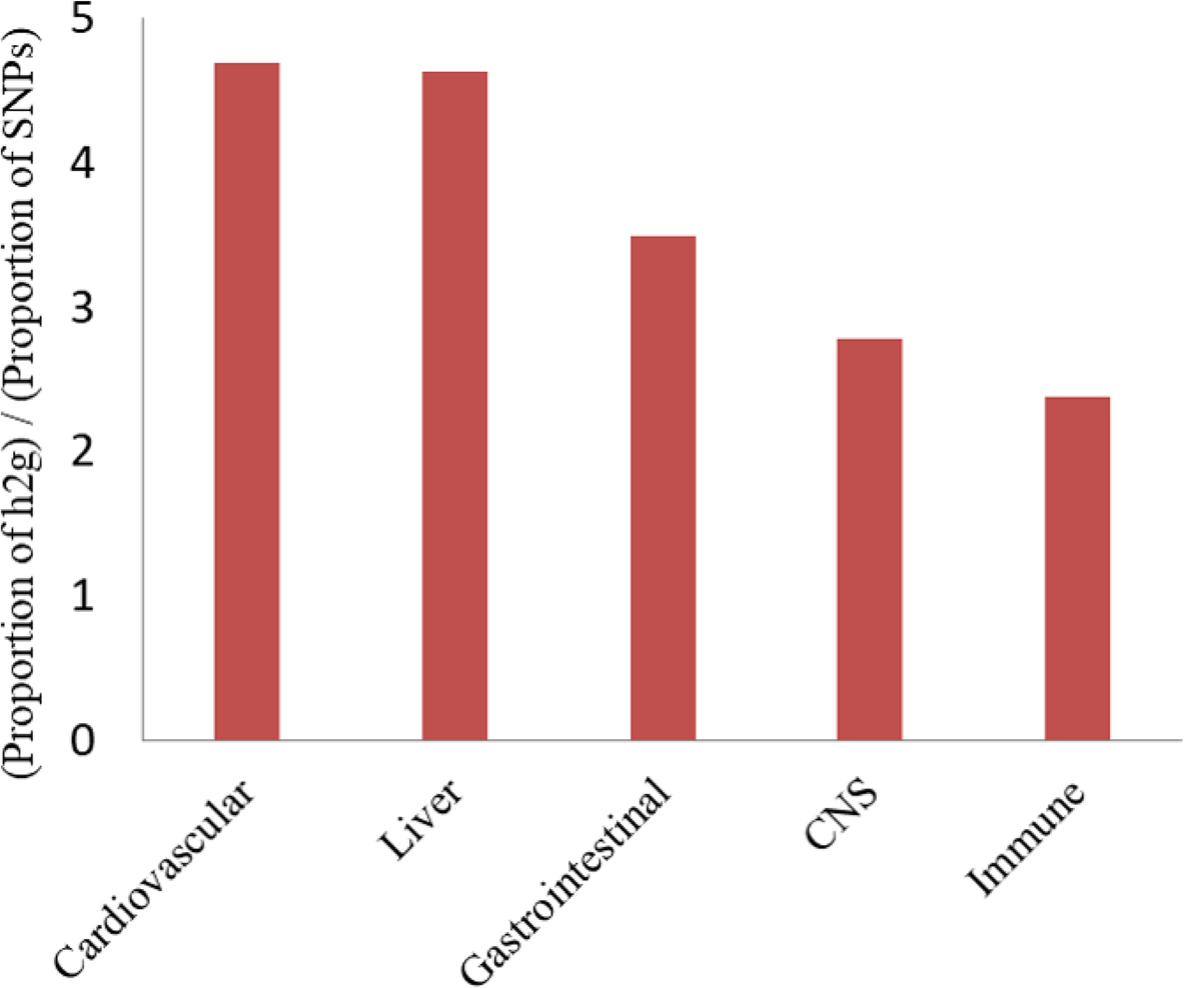
Heritability enrichment of cell type groups and SNPs and genes related to CAD functioned in cardiovascular and digestive tissues. Vertical axis represents enrichment fold that calculated by proportion of heritability divided by proportion of SNPs

**Fig. 2:**
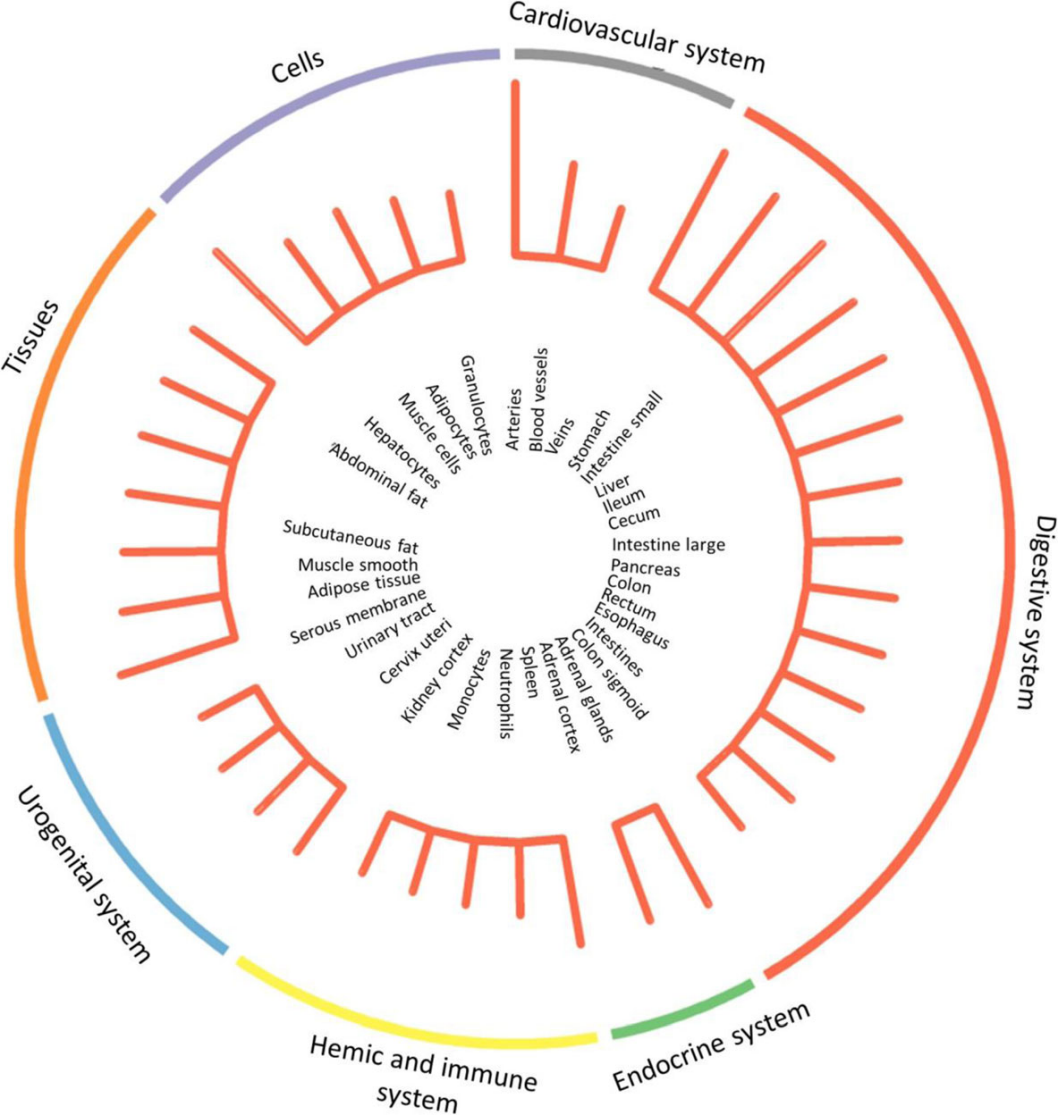
DEPICT identifies Cardiovascular and Digestive system where genes from CAD-associated loci are highly expressed. Each sector represents an organ tissue, bar length indicates the Log2(*P*-value) for that cell type or tissue

We applied stratified LD score regression to estimate the global enrichment of heritability contributed by CAD related risk SNPs in 53 genomics features annotated from 10 cell type groups. Large and significant enrichments were observed for the cardiovascular and digestive systems. For cardiovascular tissues, 11.1% SNPs explained an estimated 52.0% SNP-heritability (*P* = 1.12 × 10^− 08^ for enrichment analysis). Liver and gastrointestinal tissues showed 4.63 and 3.49 fold enrichment (*P* < 1 × 10^− 06^; Fig. 1), respectively. The significant heritability enrichment contributed by liver and gastrointestinal tissues was in line with our GWAS-based pathway analysis, highlighting that lipoprotein metabolism and cholesterol and triglyceride homeostasis contribute a genetic risk to CAD. Furthermore, DEPICT framework identified multiple tissues in the digestive system where genes from CAD-associated loci were highly expressed (Fig. 2; Additional file 2: Table S5). Although the significance level failed to pass multiple tests correction, we observed a significant enrichment of digestive system among all tissues/cell-type (14/42, Fisher’s exact *P* = 0.03).

### Genetic correlations between CAD and lipid metabolism related traits

Our pathway and functional enrichment analysis emphasized the crucial role of liver and gastrointestinal tissues in the pathology of CAD. These tissues are the main ones for producing various lipids in blood, which include low-density lipoprotein (LDL) cholesterol, high-density lipoprotein (HDL) cholesterol, triglycerides, and total cholesterol; all of them have been demonstrated to be risk factors for CAD. Thus, the shared genetic and molecular regulatory mechanisms between CAD and lipid metabolism related traits were warranted to be studied.

**Fig. 3:**
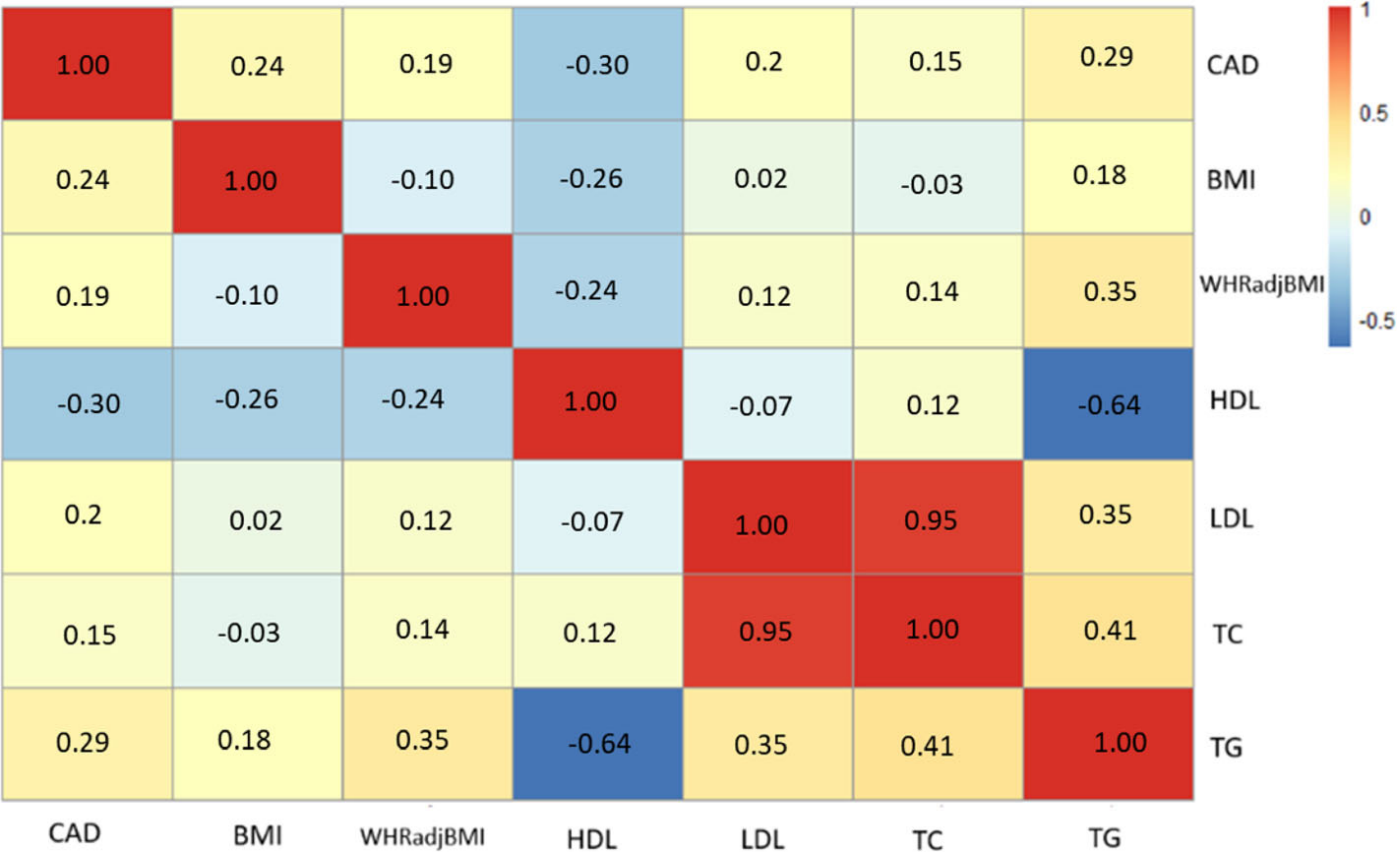
Genetic correlation between CAD and six other phenotypes (BMI, WHRadjBMI, HDL, LDL, TC, and TG). Red color represents for positive correlations and blue color represents negative correlations

We also downloaded GWAS summary data for four blood lipid level measurements from the Center for Statistical Genetics (see Methods). Moreover, BMI and waist-hip ratio GWAS data from Genetic Investigation of ANthropometric Traits (GIANT) were also included. Genetic correlations were calculated between CAD and all six traits using LD Score regression. Significant genetic correlations were found between CAD and all the other investigated traits with smallest *P* value less than 1 × 10^− 16^. We found that CAD was negatively associated with HDL-C (r = − 0.30), but showed positive correlations with LDL-C, TC, TG, BMI and WHRadjBMI (Fig. 3).

### CAD and lipid metabolism related traits shared common genetic association signals

**Fig. 4:**
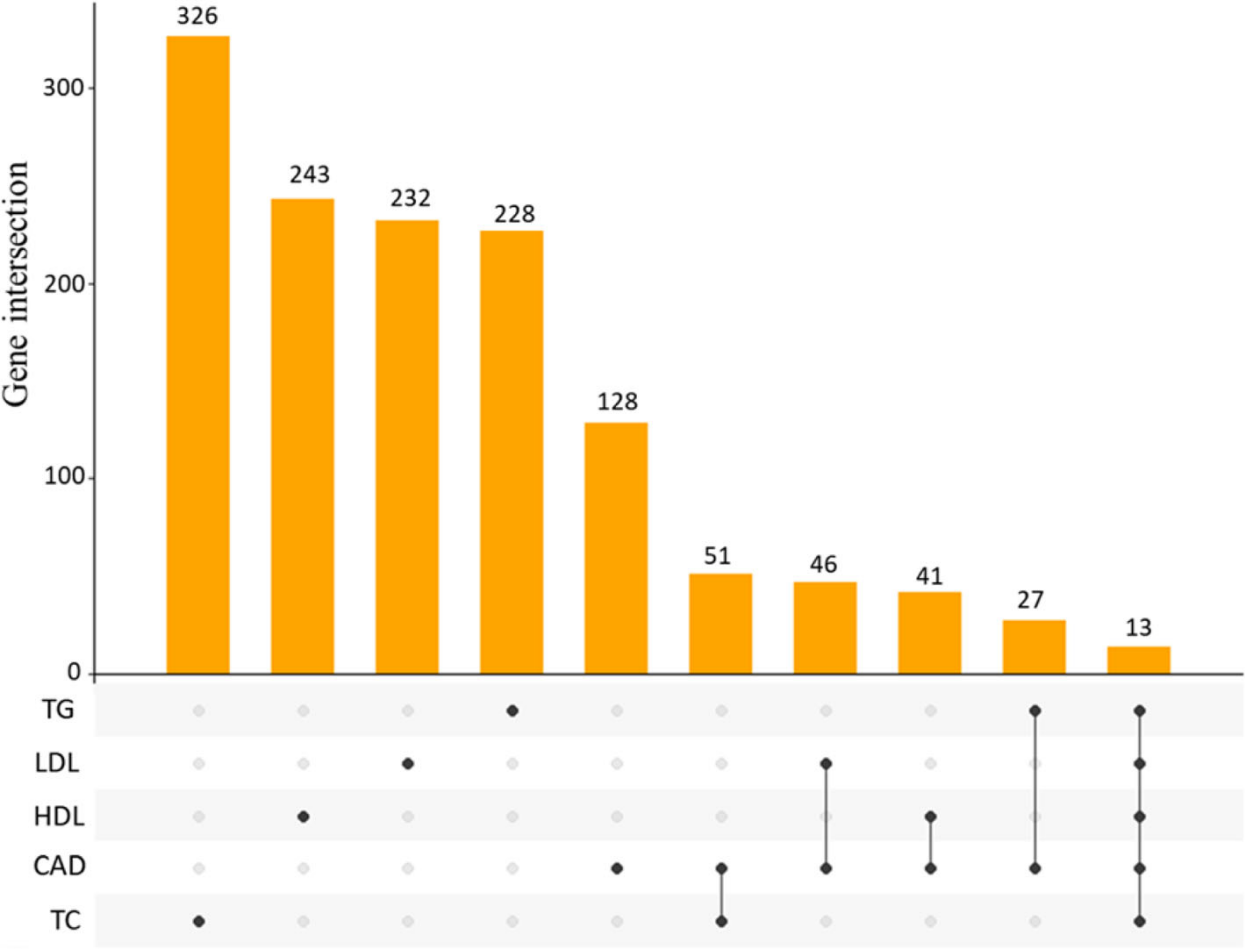
UpSetR plot shows the overlap of significantly genes discovered from MAGMA gene-based analysis between CAD and four lipid metabolism related traits

To further reveal the underlying biological mechanisms of the comorbidity between CAD and plasma lipid levels, we made a direct comparison using gene-based association signals. Gene-based association signals of genes that showed significant associations with CAD (*P* < 2.72 × 10^− 6^) were compared to that of nominally significant genes related to plasma lipid levels (*P* < 0.05, Fig. 4). We discovered that 13 genes overlapped across all five phenotypes (Gene set #1 in Additional file 2: Table S6). Notably, the common signals, including *APOC1*, *APOE*, and *APOB*, of the apolipoprotein family, which were highly expressed in the liver and played crucial roles in lipoprotein metabolism. The *PLG* gene encodes a secreted blood zymogen that is primarily expressed in liver tissue, and abnormality of this gene contributes susceptibility to thrombophilia [25].

In addition, we compared significantly CAD-enriched pathways (FDR < 0.05) with enriched pathways related to plasma lipid levels. Among these 12 top enriched pathways for CAD, 11 also showed significances among plasma lipid level traits (P < 0.05). Of them, three enriched pathways, including cholesterol homeostasis, chylomicron mediated lipid transport, and lipoprotein metabolism, maintained statistical significance after multiple testing corrections across all five traits (Table 1). Importantly, there were 6 genes (6/13) that belong to the shared Gene set #1 that also appeared in these common pathways.

**Table 1:** Common pathways between CAD and plasma lipid levels

Pathway name	P-CAD	P-HDL	P-LDL	P-TC	P-TG
Positive regulation of cholesterol storage	3.99 × 10^−09^	8.07 × 10^−22^	9.38 × 10^−03^	4.45 × 10^− 03^	2.03 × 10^− 12^
Apolipoprotein binding	1.10 × 10^−06^	8.25 × 10^−29^	1.82 × 10^−05^	1.99 × 10^− 18^	5.35 × 10^− 09^
low-density lipoprotein particle	8.32 × 10^−09^	2.13 × 10^− 05^	1.23 × 10^− 10^	8.16 × 10^− 06^	2.10 × 10^− 03^
Lipoprotein catabolic process	2.72 × 10^− 09^	9.06 × 10^− 12^	3.30 × 10^− 12^	6.44 × 10^− 11^	1.12 × 10^− 07^
Chylomicron	2.31 × 10^− 06^	3.32 × 10^−11^	6.99 × 10^− 10^	5.61 × 10^− 07^	9.59 × 10^− 21^
Cholesterol homeostasis*	9.61 × 10^− 08^	4.83 × 10^− 17^	6.99 × 10^− 15^	3.49 × 10^− 26^	4.17 × 10^− 12^
Reverse cholesterol transport	4.13 × 10^− 06^	3.76 × 10^− 23^	8.02 × 10^−05^	1.25 × 10^− 20^	2.54 × 10^− 07^
Triglyceride homeostasis	3.65 × 10^− 07^	3.32 × 10^− 18^	6.03 × 10^− 06^	5.40 × 10^− 10^	2.23 × 10^−20^
Lipase	6.16 × 10^− 07^	2.82 × 10^− 12^	2.99 × 10^− 02^	1.22 × 10^− 05^	1.77 × 10^− 08^
Chylomicron mediated lipid transport*	4.81 × 10^− 06^	6.34 × 10^− 14^	5.37 × 10^−12^	1.72 × 10^− 17^	1.16 × 10^− 18^
Lipoprotein metabolism *	7.59 × 10^− 08^	1.10 × 10^− 17^	6.29 × 10^−11^	8.01 × 10^− 19^	2.25 × 10^− 18^

The asterisk (*) marked pathways maintained significance after multiple testing corrections across all five traits

Furthermore, we sought to determine whether the 13 shared genes from gene-based analysis were significantly overrepresented in these common pathways. A random re-sampling of the same number of genes for the shared genes was conducted 10 million times. All genes within our predefined pathways (*N* = 16,994) served as the pool for our randomization test. After the randomization trials, we observed no instances of any overlap greater than the real one that contains 6 overlaps (Additional file 2: Table S7).

### Identification of co-expression modules genetically associated with CAD and blood lipid levels

**Fig. 5:**
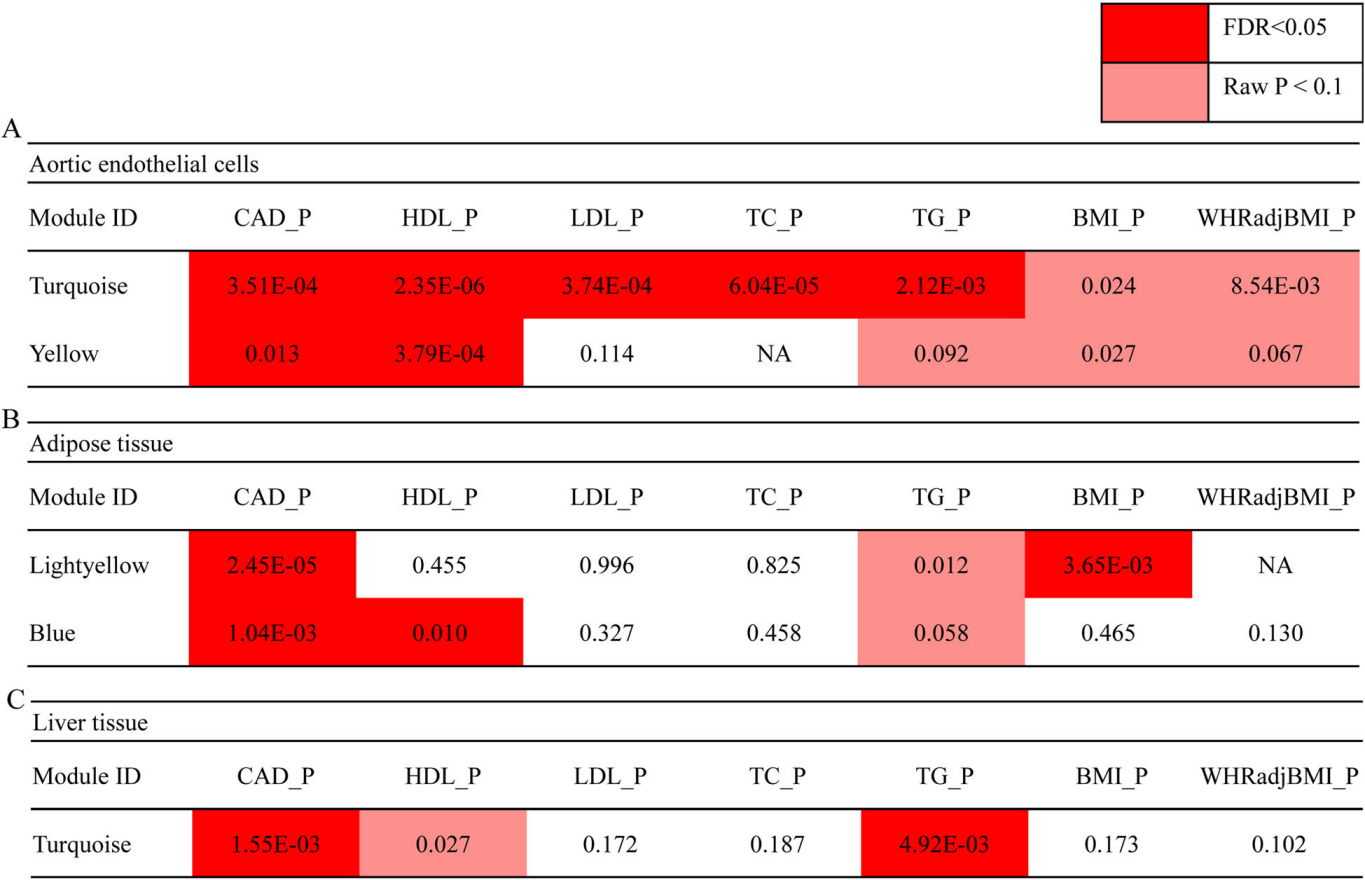
CAD associated modules show significances among lipid metabolism related traits in relevant tissues. **a** for aortic endothelial cell; **b** for adipose tissue; **c** for liver tissue. Red box corresponds FDR < 0.05 and pink box corresponds *P* value < 0.1

We investigated the expression profiles of co-expression modules that associated with genetic markers in various tissues relevant to CAD and lipid metabolism by Marker Set Enrichment Analysis (MSEA). Briefly, co-expression networks were constructed using transcriptome datasets from liver, aortic, endothelial cells and adipose, respectively (Additional file 1: Figures S1, S2, and S3). The identified modules were used as functionally related gene sets to enter into MSEA and the significance of enrichment of a co-expression module to potential functional disease SNPs defined by GWAS was assessed using Chi-square-like statistics. For each tissue or cell type, we prioritized common modules that not only significantly associated with CAD (FDR < 0.05), but also associated with at least one blood lipid traits (Fig. 5). In Aortic endothelial cells (Fig. 5a), two modules were significantly associated with CAD (P_Turquoise_ = 3.51 × 10^− 4^, P_Yellow_ = 0.013). Turquoise additionally associated with HDL (*P* = 2.35 × 10^− 6^), LDL (*P* = 3.74 × 10^− 4^), TC (*P* = 6.04 × 10^− 5^) and TG (*P* = 2.12 × 10^− 3^). In adipose tissue, a light yellow module was associated with both CAD and BMI (Fig. 5b), and a blue module was associated both CAD and HDL (Fig. 5b). One module reached significance in liver tissue (Fig. 5c).

### Prioritization of hub genes for common modules

The common co-expression module identified above mainly provided expression patterns without detailed topology information on gene-gene regulatory relationship. By applying a wKDA analysis that integrates GIANT network, we prioritized hub genes within the common modules in three relevant tissues. Together, our analysis identified 571, 2843, and 3016 significantly changed genes with FDR < 0.01 in adipose, aortic endothelial cells and liver tissue, respectively. Of them, we revealed 245 key drivers (KDs) that showed significance in all three tissues for both CAD and plasma lipid associated modules (Additional file 2: Table S8).

## Discussion

The present study systematically investigated the molecular links between CAD and plasma lipid levels by integration of GWAS signals with gene expression data. Our results showed that the genetic contribution of CAD is heavily concentrated in cell-type-specific regulatory regions of the cardiovascular and digestive systems, the sites of regulating plasma lipid level. Common genes and pathways were used to detect the effects of pleiotropy within the comorbidity between the two traits of interests. Data driven analysis of transcriptome sequences in three relevant tissues modeled co-expression networks that were significantly associated with both CAD and plasma lipids. A gene regulatory network helped to prioritized hub genes that were strongly connected in sub-networks.

Previous studies [26–28] have documented multiple lines of evidences to support the comorbidity between dyslipidemia and cardiovascular disease. The seminal finding of Framingham Heart Study showed that plasma cholesterol concentration was associated with potential CAD risk. The following randomized controlled trail (RCT) also demonstrates the causal relationship between CAD and plasma lipid levels. Evidence from human genetic studies identified loss-of-function mutations in the *LDLR* genes to be associated with high level of plasma LDL-C and premature CAD. Genes that modulate plasma triglyceride levels have been associated with CAD risk. Combined, these genetic findings suggest that lipoprotein and triglyceride-rich lipoproteins contribute to CAD. All these abovementioned gene have also been detected in our analysis, which provides an independent support to those reported findings.

In the current study, our findings provided further evidence of the underlying genetic link between CAD and plasma lipoproteins. The top enriched pathways detected in this study included lipoprotein catabolism process, positive regulation of cholesterol storage, lipoprotein metabolism, and cholesterol and triglyceride homeostasis. Notably, 8 out of 11 pathways that passed significance after corrections for multiple tests were found to be involved in the regulation of plasma lipids levels. Besides the cardiovascular system, we detected enrichments in liver and gastrointestinal tissues. In addition, DEPICT also indicated digestive system, including upper gastrointestinal tract, stomach, intestine small, hepatocytes, liver, ileum, and cecum, as the most relevant tissues where CAD risk genes highly expressed. These findings were consistent with a recent GWAS study using UK biobank samples [29].

Moreover, we found significant genetic correlations between CAD and plasma lipids based on LD score regression analysis. Our results showed that CAD was negatively correlated with HDL-C and positively correlated with LDL-C. HDL-C particles remove fats and cholesterol from cells; individuals with higher levels of HDL-C are less likely to suffer from cardiovascular diseases [30]. Inversely, LDL-C particles used as a risk factor for CAD; individuals with lower levels of LDL-C are more likely to reduce the risk of major coronary events and coronary death [31–33]. A recent epidemiology study using 4205 new-onset patients with stable CAD in Chinese population discovered that plasma HDL-C levels appear to be a predicator of coronary severity [34]. LDL-C and triglyceride-rich lipoproteins were previously treated as casual biomarkers for CAD [35].

One of the main findings in the current study pinpointed common genes and pathways implicated in the comorbidity between CAD and plasma lipids level. Our gene-based analysis found that *LDLR*, *APOB,* and *PCSK9* were significantly associated with both CAD and LDL-C. These three genes participate in the cellular LDL particle uptake, promote degradation of LDL particles, and reduce the risk of CAD [36]. The detected shared pathways unveiled by our systematic analysis included lipoprotein metabolism processes, which also provide evidence of the shared genetic vulnerability between the two traits of interests.

The systematically integrative pipeline by using multi-omics data could help us to better understand the biological mechanisms of complex diseases or comorbidities. The computational pipeline of Mergeomics combines disease-related genetic association data with pre-defined sets of connected genes to identify key drivers that are enriched for genes in the cellular regulatory network [13]. By identifying genetically-driven CAD and plasma lipids modules independently, we found that CAD related modules also exhibited significance in plasma lipids. Importantly, the CAD associated gene sets coincidently linked with plasma lipids in a tissue specific manner. The combination of univariate association signals with expression data in relevant tissues made the shared regulatory network more explicit.

By applying a comprehensive network modeling system, we identified several critical key modulators that have highly pathogenic potential for CAD. These key-drivers (KDs) were enriched in both CAD and plasma lipids associated co-expression modules, which were tissue-specific regulated. Further, we presented the sub-networks where KDs regulated many known disease genes for both CAD and plasma lipids. The gene-gene interactions or networks modules, which constructed from other independent studies, implied more comprehensive conditions that to unveil biological insights [37, 38]. We identified common KDs in three CAD-relevant tissues, indicating the crucial role of these genes implicated in the comorbidity of between CAD and plasma lipid levels.

There are several limitations in the current study. First, the data-driven analysis was constrained by the current available functional datasets. For the missing information, we expected further investigation from additional relevant tissues with multidimensional functional annotation data. Second, the inferred gene-gene interactions derived from KDs in our subnetworks need further experimental validation at various levels. The regulatory effects of KDs on neighboring genes warrant future investigation using independent in vivo and in vitro systems.

## Conclusions

In sum, the current study revealed the genetic landscape of CAD with functional enrichment of risk loci in lipoprotein metabolism processes and relevant tissues and cell types. Through integrative genetic and expression data, we identified the shared pathogenesis of CAD and plasma lipid traits, including common genes, pathways, and key molecular drivers. This systematic approach provides novel insight into basic pathogenic mechanism for cardiometabolic diseases and relevant comorbidities.

## Supplementary information

**Additional file 1: Figure S1.** Clustering dendrogram of genes for GSE30169, together with assigned module colors, **Figure S2.** Clustering dendrogram of genes for GSE7965, together with assigned module colors, **Figure S3.** Clustering dendrogram of genes for GSE24335, together with assigned module colors. (PPTX 79 kb)

**Additional file 2: Table S1.** GWAS summary association statistics used in current analysis. **Table S2** Transcriptome data used in current analysis. **Table S3.** Top pathways identified by summarized CAD GWAS data. **Table S4.** Ten genes that reached genome-wide significance in lipoprotein metabolism, cholesterol and triglyceride homeostasis pathways. **Table S5.** Tissues that showed nominal significance (*P* < 0.05) revealed by DEPICT tissue/cell type enrichment analysis. **Table S6.** 13 common genes between CAD and plasma lipid related traits. **Table S7.** Randomization results for 13 shared genes in 3 common pathways that comprising with 85 genes. **Table S8.** The identification of 245 key driver genes for both CAD and plasma lipid associated modules.

## Data Availability

Data used in the current investigation are available from public database. CAD GWAS summary data are downloaded from http://www.cardiogramplusc4d.org/data-downloads/. Plasma lipid GWAS summary data are downloaded from http://csg.sph.umich.edu/willer/public/lipids2013/. Gene expression datasets (Accession Nos. GSE30169, GSE7965, and GSE24335) are available from GEO website: https://www.ncbi.nlm.nih.gov/geo/.

## Abbreviations

CAD: Coronary artery disease
CNS: Central nervous system
DEPICT: Data-driven expression-prioritized integration for complex traits
GEO: Gene expression omnibus
GIANT: Genetic investigation of Anthropometric traits
GWAS: Genome-wide association study
HDL: High-density lipoprotein
KEGG: Kyoto encyclopedia of genes and genomes
LD: Linkage disequilibrium
LDL: Low-density lipoprotein
MAGMA: The multi-marker analysis of GenoMic annotation
MSEA: Marker set enrichment analysis
SNP: Single nucleotide polymorphism
WGCNA: The weighted correlation network analysis
wKDA: Weighted key driver analysis
