# Supplementary material for: Integrative genomic analysis identified common regulatory networks underlying the correlation between coronary artery disease and plasma lipid levels
Source: BMC Cardiovasc Disord. 2019 Dec 23;19:310. doi: 10.1186/s12872-019-01271-9 (PMC6927120; doi:10.1186/s12872-019-01271-9)
Supplement: Supplementary file 1 — Additional file 1: Figure S1. Clustering dendrogram of genes for GSE30169, together with assigned module colors, Figure S2. Clustering dendrogram of genes for GSE7965, together with assigned module colors, Figure S3. Clustering dendrogram of genes for GSE24335, together with assigned module colors. (PPTX 79 kb) [file 12872_2019_1271_MOESM1_ESM.pptx]

## Slide 1
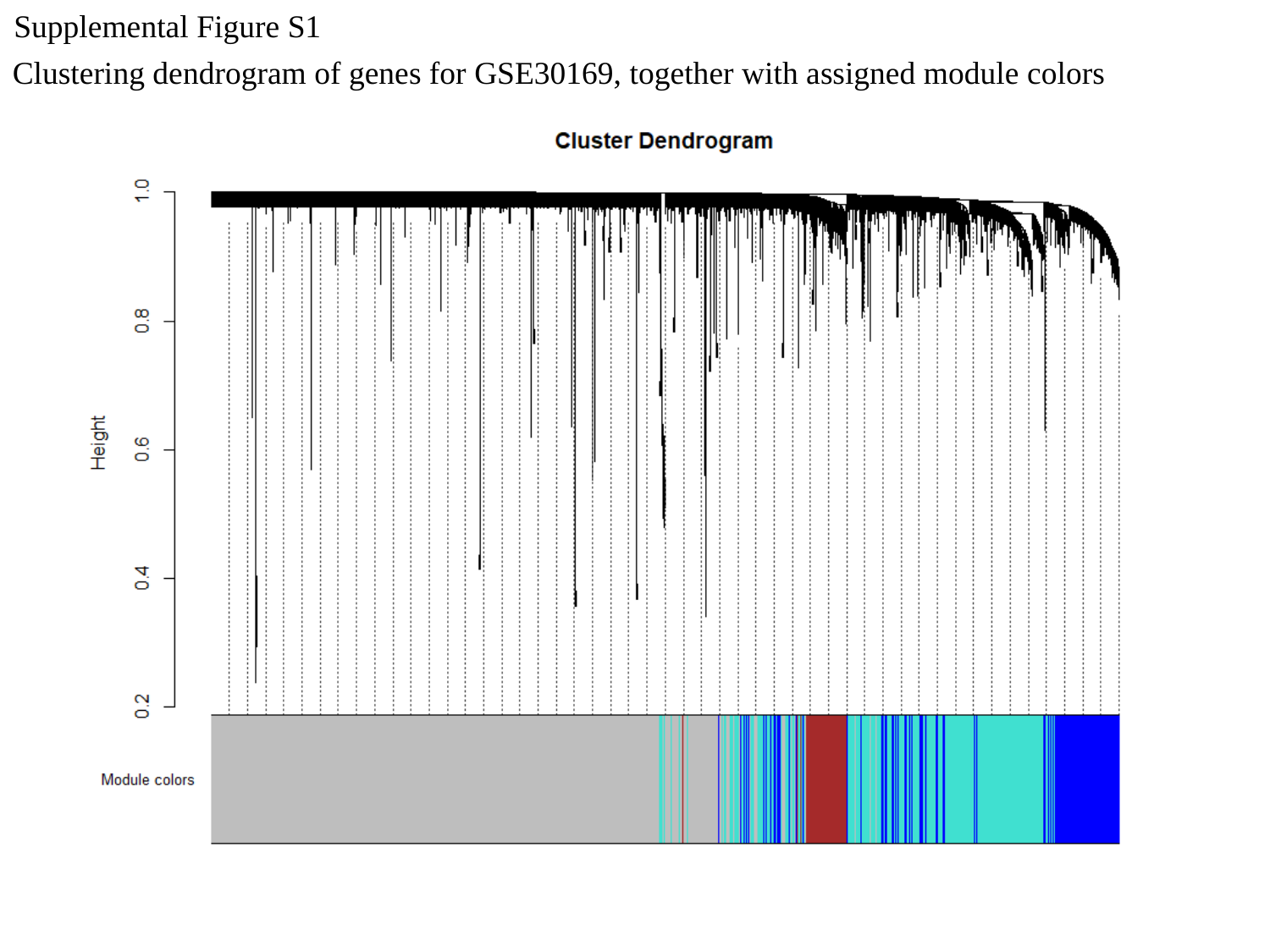

Supplemental Figure S1
Clustering dendrogram of genes for GSE30169, together with assigned module colors

## Slide 2
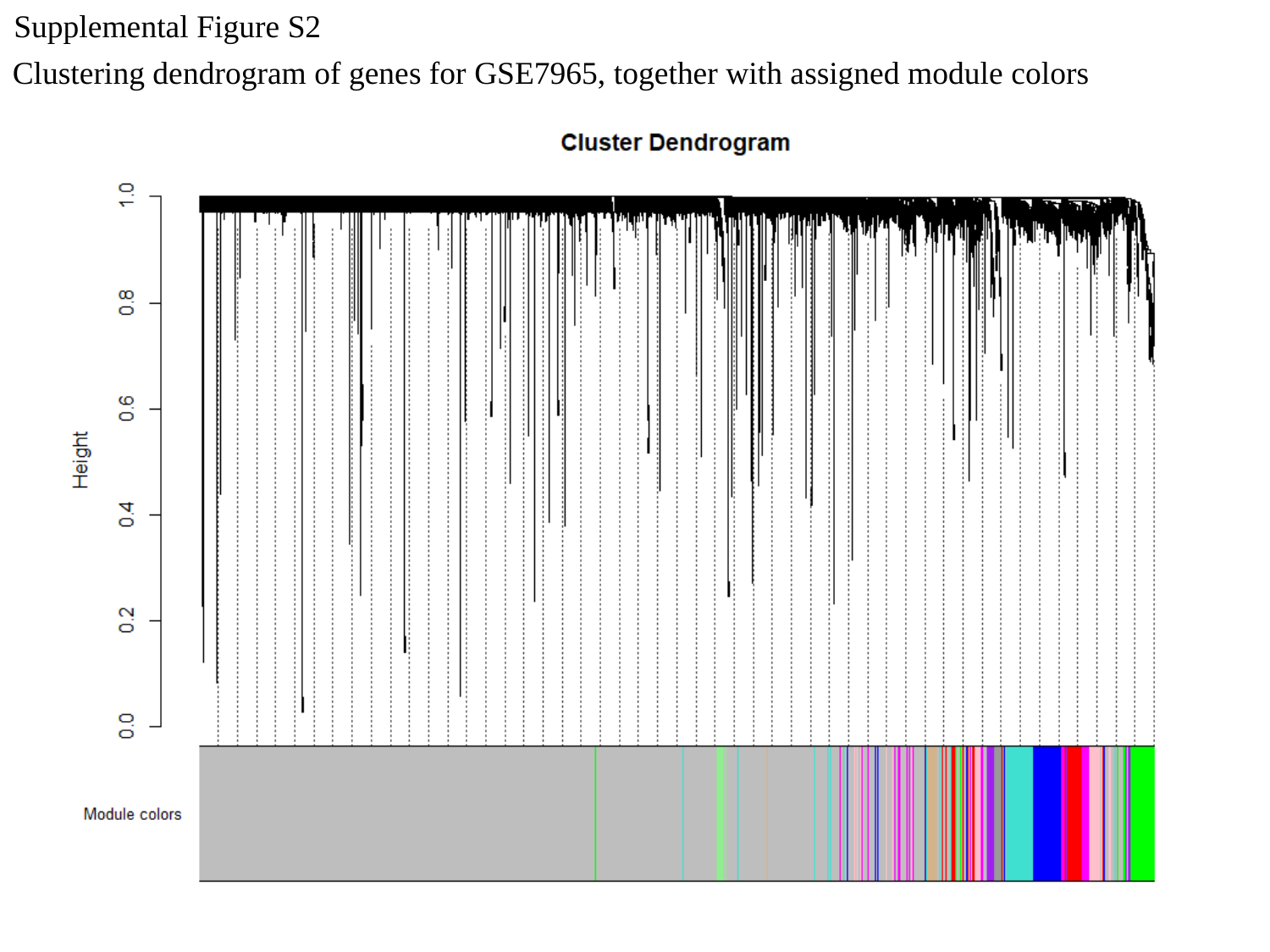

Supplemental Figure S2
Clustering dendrogram of genes for GSE7965, together with assigned module colors

## Slide 3
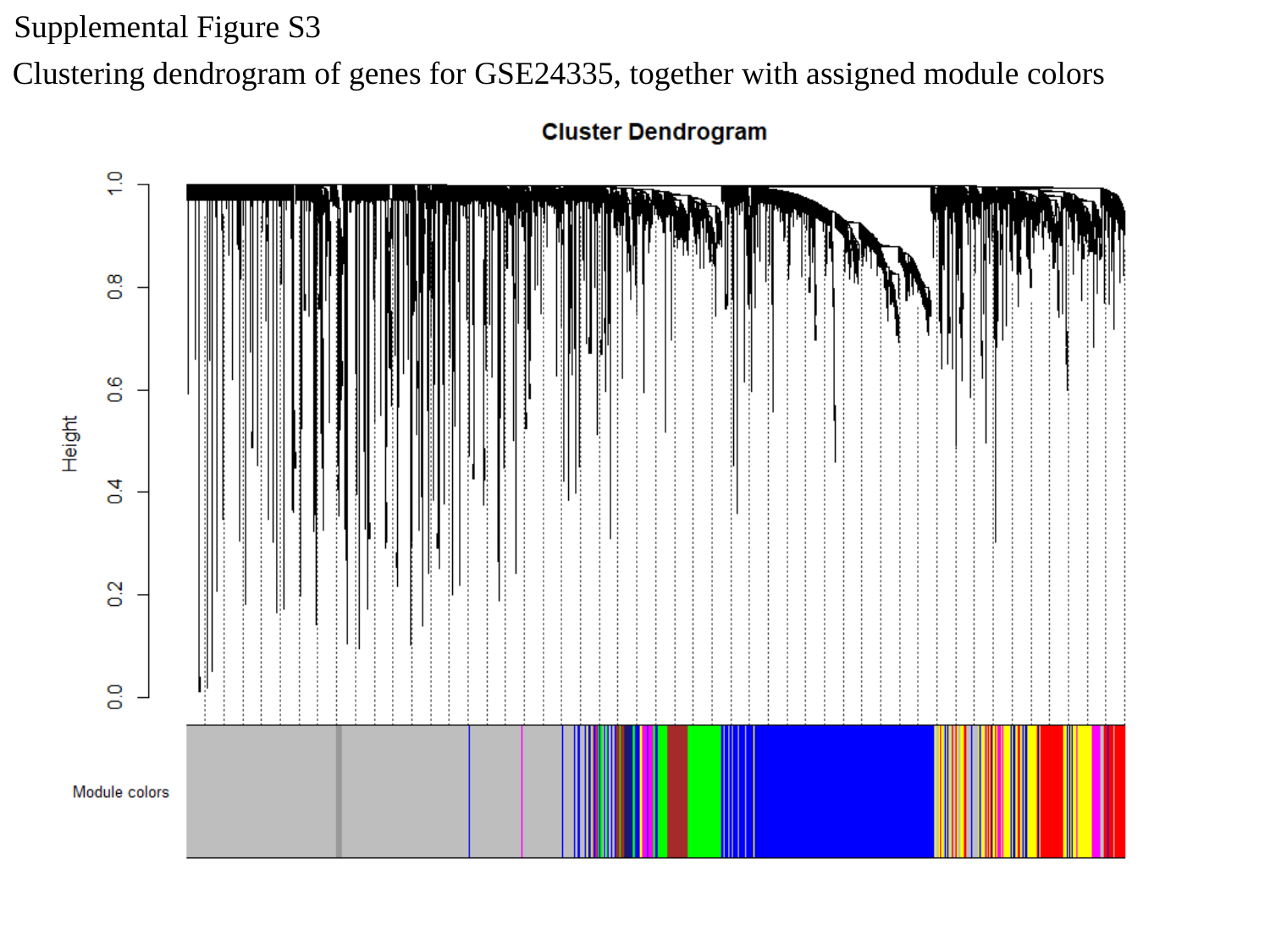

Supplemental Figure S3
Clustering dendrogram of genes for GSE24335, together with assigned module colors
