# Supplementary material for: Integrative genomic analysis identified common regulatory networks underlying the correlation between coronary artery disease and plasma lipid levels
Source: BMC Cardiovasc Disord. 2019 Dec 23;19:310. doi: 10.1186/s12872-019-01271-9 (PMC6927120; doi:10.1186/s12872-019-01271-9)
Supplement: Supplementary file 2 — Additional file 2: Table S1. GWAS summary association statistics used in current analysis. Table S2 Transcriptome data used in current analysis. Table S3. Top pathways identified by summarized CAD GWAS data. Table S4. Ten genes that reached genome-wide significance in lipoprotein metabolism, cholesterol and triglyceride homeostasis pathways. Table S5. Tissues that showed nominal significance (P < 0.05) revealed by DEPICT tissue/cell type enrichment analysis. Table S6. 13 common genes between CAD and plasma lipid related traits. Table S7. Randomization results for 13 shared genes in 3 common pathways that comprising with 85 genes. Table S8. The identification of 245 key driver genes for both CAD and plasma lipid associated modules. [file 12872_2019_1271_MOESM2_ESM.docx]

**Supplemental Tables**

**Table S1: GWAS summary association statistics used in current analysis**

| **Trait** | **Sample Size** | **References** | **URL** |
| --- | --- | --- | --- |
| Coronary artery disease   \|  \| \| --- \| | 322,183 | Nelson et al. 2017 | http://www.cardiogramplusc4d.org/data-downloads/ |
| LDL Cholesterol | 188,577 | Willer et al. 2013 | http://csg.sph.umich.edu//abecasis/public/lipids2013/ |
| HDL Cholesterol | 188,577 | Willer et al. 2013 | http://csg.sph.umich.edu//abecasis/public/lipids2013/ |
| Triglycerides | 188,577 | Willer et al. 2013 | http://csg.sph.umich.edu//abecasis/public/lipids2013/ |
| Total cholesterol TC | 188,577 | Willer et al. 2013 | http://csg.sph.umich.edu//abecasis/public/lipids2013/ |
| BMI | 339,224 | Locke et al. 2015 | http://portals.broadinstitute.org/collaboration/giant/index.php/GIANT_consortium_data_files |
| WHRadjBMI | 224,459 | Shungin et al. 2015 | http://portals.broadinstitute.org/collaboration/giant/index.php/GIANT_consortium_data_files |

Note: WHRadjBMI: waist-hip ratio adjusted for BMI

**Table S2: Transcriptome data used in current analysis**

| **GEO Accession #** | **Tissue/Cell type** | **Sample size** | **References** |
| --- | --- | --- | --- |
| GSE30169 | Aortic endothelial cells | 307 | Romanoski et al. 2011 |
| GSE7965 | Adipose tissue | 701 | Emilsson et al. 2008  Holm et al. 2010  Steinthorsdottir et al. 2014 |
| GSE24335 | Liver | 651 | Greenawalt et al. 2011  Hatoum et al, 2013 |

**Table S3: Top pathways identified by summarized CAD GWAS data**

| **Pathway name** | **Number of genes** | **Beta** | **Beta std.** | **P-value** | **Corrected P value** |
| --- | --- | --- | --- | --- | --- |
| Collagen type IV | 4 | 3.69 | 0.0543 | 1.32×10^-09^ | 0.001 |
| Lipoprotein catabolic process | 5 | 3.46 | 0.0571 | 2.72×10^-09^ | 0.002 |
| Positive regulation of cholesterol storage | 6 | 2.82 | 0.0510 | 3.99×10^-09^ | 0.002 |
| Low density lipoprotein particle | 12 | 2.00 | 0.0510 | 8.32×10^-09^ | 0.003 |
| Tissue remodeling | 5 | 2.84 | 0.0468 | 1.65×10^-08^ | 0.004 |
| Lipoprotein metabolism | 27 | 1.14 | 0.0435 | 7.59×10^-08^ | 0.010 |
| Cholesterol homeostasis | 39 | 0.861 | 0.0396 | 9.61×10^-08^ | 0.011 |
| Glomerulus development | 5 | 2.53 | 0.0418 | 3.09×10^-07^ | 0.022 |
| Triglyceride homeostasis | 9 | 1.95 | 0.0432 | 3.65×10^-07^ | 0.023 |
| Lipase | 19 | 1.24 | 0.0398 | 6.16×10^-07^ | 0.031 |
| Apolipoprotein binding | 7 | 1.85 | 0.0360 | 1.10×10^-06^ | 0.043 |
| Patterning of blood vessels | 19 | 1.16 | 0.0372 | 1.30×10^-06^ | 0.046 |

**Table S4: Ten genes that reached genome-wide significance in lipoprotein metabolism, cholesterol and triglyceride homeostasis pathways.**

| **GENE ID** | **CHR** | **START** | **STOP** | **NSNPS** | **N** | **ZSTAT** | **P** | **PERMP** | **NPERM** |
| --- | --- | --- | --- | --- | --- | --- | --- | --- | --- |
| LDLR | 19 | 11165037 | 11254506 | 340 | 327316 | 8.4979 | 9.66E-18 | 0 | 10000 |
| LPA | 6 | 1.61E+08 | 1.61E+08 | 403 | 323524 | 8.3946 | 2.34E-17 | 0 | 10000 |
| PLG | 6 | 1.61E+08 | 1.61E+08 | 304 | 321974 | 7.6502 | 1.00E-14 | 0 | 10000 |
| APOE | 19 | 45374039 | 45422650 | 160 | 318571 | 6.6976 | 1.06E-11 | 0 | 10000 |
| LIPA | 10 | 90963326 | 91046660 | 239 | 325052 | 6.6737 | 1.25E-11 | 0 | 10000 |
| LPL | 8 | 19761582 | 19834770 | 304 | 315132 | 6.0018 | 9.76E-10 | 0 | 10000 |
| APOB | 2 | 21214301 | 21301945 | 215 | 311126 | 5.4874 | 2.04E-08 | 0 | 10000 |
| ABCG8 | 2 | 44031103 | 44115947 | 467 | 325964 | 4.9622 | 3.49E-07 | 0 | 10000 |
| ABCG5 | 2 | 44029611 | 44101039 | 414 | 325922 | 4.9469 | 3.77E-07 | 0 | 10000 |
| APOC4 | 19 | 45410495 | 45458753 | 145 | 316202 | 4.9294 | 4.12E-07 | 0 | 10000 |

Note: **CHR**: the chromosome the gene is on; **START/STOP**: the annotation boundaries of the gene on that chromosome (including any window around the gene applied during annotation); **NSNPS**: the number of SNPs annotated to that gene; **N**: the sample size used when analysing that gene; **ZSTAT**: the Z-value for the gene, based on its (permutation) p-value; **P**: the gene p-value; **PERMP**: the gene p-value, using permutation-based sampling distribution; **NPERM**: the number of permutations PERMP is based on.

**Table S5: Tissues that showed nominal significance (P < 0.05) revealed by DEPICT tissue/cell type enrichment analysis.**

| **MeSH term** | **MeSH first level term** | **MeSH second level term** | **Nominal P value** | **False discovery rate < 5%** |
| --- | --- | --- | --- | --- |
| A07.231.114 | Arteries | Cardiovascular System | 2.06E-04 | No |
| A03.556.875 | Upper Gastrointestinal Tract | Digestive System | 4.76E-04 | No |
| A03.556.875.875 | Stomach | Digestive System | 7.81E-04 | No |
| A03.556.124.684 | Intestine Small | Digestive System | 1.03E-03 | No |
| A11.436.348 | Hepatocytes | Cells | 1.83E-03 | No |
| A03.620 | Liver | Digestive System | 2.03E-03 | No |
| A03.556.249.124 | Ileum | Digestive System | 2.54E-03 | No |
| A10.615.789 | Serous Membrane | Tissues | 2.67E-03 | No |
| A03.556.249.249.209 | Cecum | Digestive System | 4.02E-03 | No |
| A06.407.071 | Adrenal Glands | Endocrine System | 4.14E-03 | No |
| A15.382.520.604.700 | Spleen | Hemic and Immune Systems | 4.98E-03 | No |
| A10.165.114 | Adipose Tissue | Tissues | 6.07E-03 | No |
| A06.407.071.140 | Adrenal Cortex | Endocrine System | 6.49E-03 | No |
| A10.690.467 | Muscle Smooth | Tissues | 8.10E-03 | No |
| A10.165.114.830.750 | Subcutaneous Fat | Tissues | 8.62E-03 | No |
| A10.165.114.830 | Adipose Tissue White | Tissues | 8.62E-03 | No |
| A03.556.249 | Lower Gastrointestinal Tract | Digestive System | 8.72E-03 | No |
| A07.231 | Blood Vessels | Cardiovascular System | 8.98E-03 | No |
| A10.165.114.830.500.750 | Subcutaneous Fat Abdominal | Tissues | 9.04E-03 | No |
| A10.165.114.830.500 | Abdominal Fat | Tissues | 9.04E-03 | No |
| A02.165 | Cartilage | Musculoskeletal System | 0.010 | No |
| A03.556.249.249 | Intestine Large | Digestive System | 0.011 | No |
| A03.734 | Pancreas | Digestive System | 0.012 | No |
| A03.556.249.249.356 | Colon | Digestive System | 0.012 | No |
| A11.620.520 | Myocytes Smooth Muscle | Cells | 0.013 | No |
| A11.620 | Muscle Cells | Cells | 0.013 | No |
| A03.556.124.526.767 | Rectum | Digestive System | 0.013 | No |
| A03.556.875.500 | Esophagus | Digestive System | 0.015 | No |
| A04.411 | Lung | Respiratory System | 0.019 | No |
| A03.556.124 | Intestines | Digestive System | 0.019 | No |
| A05.810.453.324 | Kidney Cortex | Urogenital System | 0.020 | No |
| A11.329.114 | Adipocytes | Cells | 0.024 | No |
| A05.360.319.679.690 | Myometrium | Urogenital System | 0.026 | No |
| A15.382.490.315.583 | Neutrophils | Hemic and Immune Systems | 0.028 | No |
| A05.360.319.679.256 | Cervix Uteri | Urogenital System | 0.028 | No |
| A11.118.637.415 | Granulocytes | Cells | 0.036 | No |
| A15.378.316.580 | Monocytes | Hemic and Immune Systems | 0.040 | No |
| A03.556.249.249.356.668 | Colon Sigmoid | Digestive System | 0.040 | No |
| A15.378.316 | Bone Marrow Cells | Hemic and Immune Systems | 0.042 | No |
| A15.378 | Hematopoietic System | Hemic and Immune Systems | 0.042 | No |
| A07.231.908 | Veins | Cardiovascular System | 0.046 | No |
| A05.810 | Urinary Tract | Urogenital System | 0.048 | No |

Note: MeSH term: Medical Subject Heading term for the tissue or cell type annotation; MeSH first level term: Description of the tissue or cell type annotation; MeSH second level term: More general description of the tissue or cell type annotation.

**Table S6: 13 common genes between CAD and plasma lipid related traits**

| **Gene name** | **P-CAD** | **P-HDL** | **P-LDL** | **P-TC** | **P-TG** |
| --- | --- | --- | --- | --- | --- |
| *LPA* | 2.34×10^-17^ | 2.55×10^-06^ | 1.16×10^-12^ | 1.76×10^-11^ | 5.09×10^-04^ |
| *APOC1* | 1.29×10^-12^ | 1.80×10^-14^ | 1.31×10^-71^ | 5.12×10^-64^ | 6.72×10^-33^ |
| *APOE* | 1.06×10^-11^ | 7.68×10^-14^ | 2.35×10^-66^ | 4.69×10^-60^ | 8.15×10^-34^ |
| *SLC22A3* | 3.00×10^-11^ | 2.07×10^-02^ | 6.28×10^-08^ | 3.11×10^-10^ | 5.51×10^-08^ |
| *TOMM40* | 1.70×10^-09^ | 1.62×10^-13^ | 2.06×10^-77^ | 5.87×10^-67^ | 1.25×10^-34^ |
| *C19orf38* | 2.17×10^-09^ | 1.14×10^-04^ | 2.16×10^-20^ | 1.62×10^-15^ | 1.81×10^-02^ |
| *APOB* | 2.04×10^-08^ | 2.19×10^-18^ | 5.51×10^-107^ | 1.52×10^-95^ | 2.64×10^-20^ |
| *PVRL2* | 6.91×10^-08^ | 1.14×10^-12^ | 8.86×10^-98^ | 2.59×10^-81^ | 3.94×10^-22^ |
| *IGF2R* | 3.24×10^-07^ | 1.57×10^-04^ | 1.87×10^-11^ | 1.76×10^-11^ | 2.81×10^-03^ |
| *APOC4* | 4.12×10^-07^ | 1.45×10^-13^ | 2.43×10^-25^ | 6.64×10^-23^ | 1.14×10^-12^ |
| *SNRPD2* | 6.44×10^-07^ | 5.39×10^-04^ | 5.26×10^-06^ | 2.22E-06 | 4.70×10^-05^ |
| *DNM2* | 1.76×10^-06^ | 3.98×10^-05^ | 3.20×10^-16^ | 7.64×10^-14^ | 8.50×10^-03^ |

**Table S7: Randomization results for 13 shared genes in 3 common pathways that comprising with 85 genes.**

| Number of overlaps | 0 | 1 | 2 | 3 | 4 | 5 | 6 |
| --- | --- | --- | --- | --- | --- | --- | --- |
| Times | 93683701 | 6129786 | 183189 | 3288 | 36 | 0 | 0 |

**Table S8: The identification of 245 key driver genes for both CAD and plasma lipid associated modules**

| *PTGR1* | prostaglandin reductase 1 [Source:HGNC Symbol;Acc:HGNC:18429] |
| --- | --- |
| *CBWD3* | COBW domain containing 3 [Source:HGNC Symbol;Acc:HGNC:18519] |
| *AK4* | adenylate kinase 4 [Source:HGNC Symbol;Acc:HGNC:363] |
| *ASPH* | aspartate beta-hydroxylase [Source:HGNC Symbol;Acc:HGNC:757] |
| *PALM2-AKAP2* | PALM2-AKAP2 readthrough [Source:HGNC Symbol;Acc:HGNC:33529] |
| *NBPF10* | neuroblastoma breakpoint family member 10 [Source:HGNC Symbol;Acc:HGNC:31992] |
| *DHX33* | DEAH-box helicase 33 [Source:HGNC Symbol;Acc:HGNC:16718] |
| *GRPEL2* | GrpE like 2, mitochondrial [Source:HGNC Symbol;Acc:HGNC:21060] |
| *FAM83D* | family with sequence similarity 83 member D [Source:HGNC Symbol;Acc:HGNC:16122] |
| *FNDC3B* | fibronectin type III domain containing 3B [Source:HGNC Symbol;Acc:HGNC:24670] |
| *MSANTD3* | Myb/SANT DNA binding domain containing 3 [Source:HGNC Symbol;Acc:HGNC:23370] |
| *LYSMD2* | LysM domain containing 2 [Source:HGNC Symbol;Acc:HGNC:28571] |
| *ARL6IP6* | ADP ribosylation factor like GTPase 6 interacting protein 6 [Source:HGNC Symbol;Acc:HGNC:24048] |
| *ARHGAP18* | Rho GTPase activating protein 18 [Source:HGNC Symbol;Acc:HGNC:21035] |
| *EXOC6* | exocyst complex component 6 [Source:HGNC Symbol;Acc:HGNC:23196] |
| *ANLN* | anillin actin binding protein [Source:HGNC Symbol;Acc:HGNC:14082] |
| *BLOC1S6* | biogenesis of lysosomal organelles complex 1 subunit 6 [Source:HGNC Symbol;Acc:HGNC:8549] |
| *RAB4A* | RAB4A, member RAS oncogene family [Source:HGNC Symbol;Acc:HGNC:9781] |
| *C14orf142* | chromosome 14 open reading frame 142 [Source:HGNC Symbol;Acc:HGNC:20356] |
| *SCOC* | short coiled-coil protein [Source:HGNC Symbol;Acc:HGNC:20335] |
| *GFM2* | G elongation factor, mitochondrial 2 [Source:HGNC Symbol;Acc:HGNC:29682] |
| *SURF4* | surfeit 4 [Source:HGNC Symbol;Acc:HGNC:11476] |
| *UCK2* | uridine-cytidine kinase 2 [Source:HGNC Symbol;Acc:HGNC:12562] |
| *OSTC* | oligosaccharyltransferase complex subunit (non-catalytic) [Source:HGNC Symbol;Acc:HGNC:24448] |
| *RPL10* | ribosomal protein L10 [Source:HGNC Symbol;Acc:HGNC:10298] |
| *PDGFC* | platelet derived growth factor C [Source:HGNC Symbol;Acc:HGNC:8801] |
| *YIPF5* | Yip1 domain family member 5 [Source:HGNC Symbol;Acc:HGNC:24877] |
| *DRAM1* | DNA damage regulated autophagy modulator 1 [Source:HGNC Symbol;Acc:HGNC:25645] |
| *TRMT6* | tRNA methyltransferase 6 [Source:HGNC Symbol;Acc:HGNC:20900] |
| *DIRC2* | disrupted in renal carcinoma 2 [Source:HGNC Symbol;Acc:HGNC:16628] |
| *NFKBIZ* | NFKB inhibitor zeta [Source:HGNC Symbol;Acc:HGNC:29805] |
| *FBXW2* | F-box and WD repeat domain containing 2 [Source:HGNC Symbol;Acc:HGNC:13608] |
| *NUFIP2* | NUFIP2, FMR1 interacting protein 2 [Source:HGNC Symbol;Acc:HGNC:17634] |
| *SLC39A10* | solute carrier family 39 member 10 [Source:HGNC Symbol;Acc:HGNC:20861] |
| *DCUN1D5* | defective in cullin neddylation 1 domain containing 5 [Source:HGNC Symbol;Acc:HGNC:28409] |
| *HIGD1A* | HIG1 hypoxia inducible domain family member 1A [Source:HGNC Symbol;Acc:HGNC:29527] |
| *REXO2* | RNA exonuclease 2 [Source:HGNC Symbol;Acc:HGNC:17851] |
| *RANBP9* | RAN binding protein 9 [Source:HGNC Symbol;Acc:HGNC:13727] |
| *RAB7A* | RAB7A, member RAS oncogene family [Source:HGNC Symbol;Acc:HGNC:9788] |
| *SOD2* | superoxide dismutase 2, mitochondrial [Source:HGNC Symbol;Acc:HGNC:11180] |
| *GABPB1* | GA binding protein transcription factor beta subunit 1 [Source:HGNC Symbol;Acc:HGNC:4074] |
| *DST* | dystonin [Source:HGNC Symbol;Acc:HGNC:1090] |
| *DAB2* | DAB2, clathrin adaptor protein [Source:HGNC Symbol;Acc:HGNC:2662] |
| *LDLR* | low density lipoprotein receptor [Source:HGNC Symbol;Acc:HGNC:6547] |
| *ENSA* | endosulfine alpha [Source:HGNC Symbol;Acc:HGNC:3360] |
| *SERPINE2* | serpin family E member 2 [Source:HGNC Symbol;Acc:HGNC:8951] |
| *CTTN* | cortactin [Source:HGNC Symbol;Acc:HGNC:3338] |
| *BTG3* | BTG family member 3 [Source:HGNC Symbol;Acc:HGNC:1132] |
| *CYLD* | CYLD lysine 63 deubiquitinase [Source:HGNC Symbol;Acc:HGNC:2584] |
| *IDS* | iduronate 2-sulfatase [Source:HGNC Symbol;Acc:HGNC:5389] |
| *GRB10* | growth factor receptor bound protein 10 [Source:HGNC Symbol;Acc:HGNC:4564] |
| *RBPMS* | RNA binding protein with multiple splicing [Source:HGNC Symbol;Acc:HGNC:19097] |
| *REEP5* | receptor accessory protein 5 [Source:HGNC Symbol;Acc:HGNC:30077] |
| *YTHDC1* | YTH domain containing 1 [Source:HGNC Symbol;Acc:HGNC:30626] |
| *EMP1* | epithelial membrane protein 1 [Source:HGNC Symbol;Acc:HGNC:3333] |
| *ANAPC5* | anaphase promoting complex subunit 5 [Source:HGNC Symbol;Acc:HGNC:15713] |
| *TAPBP* | TAP binding protein (tapasin) [Source:HGNC Symbol;Acc:HGNC:11566] |
| *RIPK2* | receptor interacting serine/threonine kinase 2 [Source:HGNC Symbol;Acc:HGNC:10020] |
| *MPZL1* | myelin protein zero like 1 [Source:HGNC Symbol;Acc:HGNC:7226] |
| *TFPI* | tissue factor pathway inhibitor [Source:HGNC Symbol;Acc:HGNC:11760] |
| *TUBB* | tubulin beta class I [Source:HGNC Symbol;Acc:HGNC:20778] |
| *HK2* | hexokinase 2 [Source:HGNC Symbol;Acc:HGNC:4923] |
| *ITGB1BP1* | integrin subunit beta 1 binding protein 1 [Source:HGNC Symbol;Acc:HGNC:23927] |
| *KYNU* | kynureninase [Source:HGNC Symbol;Acc:HGNC:6469] |
| *CAV2* | caveolin 2 [Source:HGNC Symbol;Acc:HGNC:1528] |
| *NUP50* | nucleoporin 50 [Source:HGNC Symbol;Acc:HGNC:8065] |
| *PDXK* | pyridoxal (pyridoxine, vitamin B6) kinase [Source:HGNC Symbol;Acc:HGNC:8819] |
| *MATR3* | matrin 3 [Source:HGNC Symbol;Acc:HGNC:6912] |
| *ABCC1* | ATP binding cassette subfamily C member 1 [Source:HGNC Symbol;Acc:HGNC:51] |
| *NUP62* | nucleoporin 62 [Source:HGNC Symbol;Acc:HGNC:8066] |
| *EXOSC7* | exosome component 7 [Source:HGNC Symbol;Acc:HGNC:28112] |
| *PHLDA1* | pleckstrin homology like domain family A member 1 [Source:HGNC Symbol;Acc:HGNC:8933] |
| *LRP10* | LDL receptor related protein 10 [Source:HGNC Symbol;Acc:HGNC:14553] |
| *TSPAN4* | tetraspanin 4 [Source:HGNC Symbol;Acc:HGNC:11859] |
| *TM4SF1* | transmembrane 4 L six family member 1 [Source:HGNC Symbol;Acc:HGNC:11853] |
| *SFPQ* | splicing factor proline and glutamine rich [Source:HGNC Symbol;Acc:HGNC:10774] |
| *AHNAK* | AHNAK nucleoprotein [Source:HGNC Symbol;Acc:HGNC:347] |
| *YPEL5* | yippee like 5 [Source:HGNC Symbol;Acc:HGNC:18329] |
| *UBE2L3* | ubiquitin conjugating enzyme E2 L3 [Source:HGNC Symbol;Acc:HGNC:12488] |
| *YWHAG* | tyrosine 3-monooxygenase/tryptophan 5-monooxygenase activation protein gamma [Source:HGNC Symbol;Acc:HGNC:12852] |
| *DSTN* | destrin, actin depolymerizing factor [Source:HGNC Symbol;Acc:HGNC:15750] |
| *SERBP1* | SERPINE1 mRNA binding protein 1 [Source:HGNC Symbol;Acc:HGNC:17860] |
| *WWTR1* | WW domain containing transcription regulator 1 [Source:HGNC Symbol;Acc:HGNC:24042] |
| *KDM5B* | lysine demethylase 5B [Source:HGNC Symbol;Acc:HGNC:18039] |
| *RAB27A* | RAB27A, member RAS oncogene family [Source:HGNC Symbol;Acc:HGNC:9766] |
| *TRIM2* | tripartite motif containing 2 [Source:HGNC Symbol;Acc:HGNC:15974] |
| *ANP32E* | acidic nuclear phosphoprotein 32 family member E [Source:HGNC Symbol;Acc:HGNC:16673] |
| *CLIC4* | chloride intracellular channel 4 [Source:HGNC Symbol;Acc:HGNC:13518] |
| *RBMS1* | RNA binding motif single stranded interacting protein 1 [Source:HGNC Symbol;Acc:HGNC:9907] |
| *SEC24A* | SEC24 homolog A, COPII coat complex component [Source:HGNC Symbol;Acc:HGNC:10703] |
| *MAP1LC3B* | microtubule associated protein 1 light chain 3 beta [Source:HGNC Symbol;Acc:HGNC:13352] |
| *VDAC1* | voltage dependent anion channel 1 [Source:HGNC Symbol;Acc:HGNC:12669] |
| *NQO1* | NAD(P)H quinone dehydrogenase 1 [Source:HGNC Symbol;Acc:HGNC:2874] |
| *BUB3* | BUB3, mitotic checkpoint protein [Source:HGNC Symbol;Acc:HGNC:1151] |
| *TCF3* | transcription factor 3 [Source:HGNC Symbol;Acc:HGNC:11633] |
| *ACTR1A* | ARP1 actin-related protein 1 homolog A, centractin alpha [Source:HGNC Symbol;Acc:HGNC:167] |
| *ARL6IP5* | ADP ribosylation factor like GTPase 6 interacting protein 5 [Source:HGNC Symbol;Acc:HGNC:16937] |
| *DIMT1* | DIM1 dimethyladenosine transferase 1 homolog [Source:HGNC Symbol;Acc:HGNC:30217] |
| *TSPAN3* | tetraspanin 3 [Source:HGNC Symbol;Acc:HGNC:17752] |
| *CPD* | carboxypeptidase D [Source:HGNC Symbol;Acc:HGNC:2301] |
| *MAGED2* | MAGE family member D2 [Source:HGNC Symbol;Acc:HGNC:16353] |
| *BNIP3L* | BCL2/adenovirus E1B 19kDa interacting protein 3-like [Source:HGNC Symbol;Acc:HGNC:1085] |
| *ANXA2* | annexin A2 [Source:HGNC Symbol;Acc:HGNC:537] |
| *CFLAR* | CASP8 and FADD like apoptosis regulator [Source:HGNC Symbol;Acc:HGNC:1876] |
| *GDI2* | GDP dissociation inhibitor 2 [Source:HGNC Symbol;Acc:HGNC:4227] |
| *SSR1* | signal sequence receptor subunit 1 [Source:HGNC Symbol;Acc:HGNC:11323] |
| *AP1S2* | adaptor related protein complex 1 sigma 2 subunit [Source:HGNC Symbol;Acc:HGNC:560] |
| *SRPRA* | SRP receptor alpha subunit [Source:HGNC Symbol;Acc:HGNC:11307] |
| *PTBP1* | polypyrimidine tract binding protein 1 [Source:HGNC Symbol;Acc:HGNC:9583] |
| *TPM4* | tropomyosin 4 [Source:HGNC Symbol;Acc:HGNC:12013] |
| *P4HB* | prolyl 4-hydroxylase subunit beta [Source:HGNC Symbol;Acc:HGNC:8548] |
| *SP100* | SP100 nuclear antigen [Source:HGNC Symbol;Acc:HGNC:11206] |
| *EI24* | EI24, autophagy associated transmembrane protein [Source:HGNC Symbol;Acc:HGNC:13276] |
| *UBE2N* | ubiquitin conjugating enzyme E2 N [Source:HGNC Symbol;Acc:HGNC:12492] |
| *SAT1* | spermidine/spermine N1-acetyltransferase 1 [Source:HGNC Symbol;Acc:HGNC:10540] |
| *ANXA11* | annexin A11 [Source:HGNC Symbol;Acc:HGNC:535] |
| *TMBIM6* | transmembrane BAX inhibitor motif containing 6 [Source:HGNC Symbol;Acc:HGNC:11723] |
| *STMN1* | stathmin 1 [Source:HGNC Symbol;Acc:HGNC:6510] |
| *DNAJB6* | DnaJ heat shock protein family (Hsp40) member B6 [Source:HGNC Symbol;Acc:HGNC:14888] |
| *DHFR* | dihydrofolate reductase [Source:HGNC Symbol;Acc:HGNC:2861] |
| *CTSB* | cathepsin B [Source:HGNC Symbol;Acc:HGNC:2527] |
| *GALNT1* | polypeptide N-acetylgalactosaminyltransferase 1 [Source:HGNC Symbol;Acc:HGNC:4123] |
| *LITAF* | lipopolysaccharide induced TNF factor [Source:HGNC Symbol;Acc:HGNC:16841] |
| *SRGN* | serglycin [Source:HGNC Symbol;Acc:HGNC:9361] |
| *CALD1* | caldesmon 1 [Source:HGNC Symbol;Acc:HGNC:1441] |
| *EIF4B* | eukaryotic translation initiation factor 4B [Source:HGNC Symbol;Acc:HGNC:3285] |
| *AK2* | adenylate kinase 2 [Source:HGNC Symbol;Acc:HGNC:362] |
| *ENC1* | ectodermal-neural cortex 1 [Source:HGNC Symbol;Acc:HGNC:3345] |
| *TPM1* | tropomyosin 1 (alpha) [Source:HGNC Symbol;Acc:HGNC:12010] |
| *MAP4K4* | mitogen-activated protein kinase kinase kinase kinase 4 [Source:HGNC Symbol;Acc:HGNC:6866] |
| *TYMS* | thymidylate synthetase [Source:HGNC Symbol;Acc:HGNC:12441] |
| *MAGED1* | MAGE family member D1 [Source:HGNC Symbol;Acc:HGNC:6813] |
| *CALU* | calumenin [Source:HGNC Symbol;Acc:HGNC:1458] |
| *ALDH2* | aldehyde dehydrogenase 2 family (mitochondrial) [Source:HGNC Symbol;Acc:HGNC:404] |
| *FDFT1* | farnesyl-diphosphate farnesyltransferase 1 [Source:HGNC Symbol;Acc:HGNC:3629] |
| *CTNNA1* | catenin alpha 1 [Source:HGNC Symbol;Acc:HGNC:2509] |
| *SNU13* | SNU13 homolog, small nuclear ribonucleoprotein (U4/U6.U5) [Source:HGNC Symbol;Acc:HGNC:7819] |
| *CKAP4* | cytoskeleton-associated protein 4 [Source:HGNC Symbol;Acc:HGNC:16991] |
| *PWP1* | PWP1 homolog, endonuclein [Source:HGNC Symbol;Acc:HGNC:17015] |
| *KLF6* | Kruppel like factor 6 [Source:HGNC Symbol;Acc:HGNC:2235] |
| *P4HA1* | prolyl 4-hydroxylase subunit alpha 1 [Source:HGNC Symbol;Acc:HGNC:8546] |
| *IGFBP7* | insulin like growth factor binding protein 7 [Source:HGNC Symbol;Acc:HGNC:5476] |
| *PTEN* | phosphatase and tensin homolog [Source:HGNC Symbol;Acc:HGNC:9588] |
| *TCF4* | transcription factor 4 [Source:HGNC Symbol;Acc:HGNC:11634] |
| *PLOD2* | procollagen-lysine,2-oxoglutarate 5-dioxygenase 2 [Source:HGNC Symbol;Acc:HGNC:9082] |
| *DEGS1* | delta(4)-desaturase, sphingolipid 1 [Source:HGNC Symbol;Acc:HGNC:13709] |
| *RPL31* | ribosomal protein L31 [Source:HGNC Symbol;Acc:HGNC:10334] |
| *PTP4A1* | protein tyrosine phosphatase type IVA, member 1 [Source:HGNC Symbol;Acc:HGNC:9634] |
| *CD44* | CD44 molecule (Indian blood group) [Source:HGNC Symbol;Acc:HGNC:1681] |
| *TFRC* | transferrin receptor [Source:HGNC Symbol;Acc:HGNC:11763] |
| *CAT* | catalase [Source:HGNC Symbol;Acc:HGNC:1516] |
| *MAPRE1* | microtubule associated protein RP/EB family member 1 [Source:HGNC Symbol;Acc:HGNC:6890] |
| *PRDX3* | peroxiredoxin 3 [Source:HGNC Symbol;Acc:HGNC:9354] |
| *VEGFA* | vascular endothelial growth factor A [Source:HGNC Symbol;Acc:HGNC:12680] |
| *LAMB1* | laminin subunit beta 1 [Source:HGNC Symbol;Acc:HGNC:6486] |
| *CTSC* | cathepsin C [Source:HGNC Symbol;Acc:HGNC:2528] |
| *SQSTM1* | sequestosome 1 [Source:HGNC Symbol;Acc:HGNC:11280] |
| *ALCAM* | activated leukocyte cell adhesion molecule [Source:HGNC Symbol;Acc:HGNC:400] |
| *CCND1* | cyclin D1 [Source:HGNC Symbol;Acc:HGNC:1582] |
| *BCL6* | B-cell CLL/lymphoma 6 [Source:HGNC Symbol;Acc:HGNC:1001] |
| *INSIG1* | insulin induced gene 1 [Source:HGNC Symbol;Acc:HGNC:6083] |
| *FTH1* | ferritin heavy chain 1 [Source:HGNC Symbol;Acc:HGNC:3976] |
| *LMNA* | lamin A/C [Source:HGNC Symbol;Acc:HGNC:6636] |
| *MARCKS* | myristoylated alanine rich protein kinase C substrate [Source:HGNC Symbol;Acc:HGNC:6759] |
| *MCL1* | myeloid cell leukemia 1 [Source:HGNC Symbol;Acc:HGNC:6943] |
| *DUSP1* | dual specificity phosphatase 1 [Source:HGNC Symbol;Acc:HGNC:3064] |
| *EIF4G1* | eukaryotic translation initiation factor 4 gamma 1 [Source:HGNC Symbol;Acc:HGNC:3296] |
| *SRSF3* | serine and arginine rich splicing factor 3 [Source:HGNC Symbol;Acc:HGNC:10785] |
| *UGCG* | UDP-glucose ceramide glucosyltransferase [Source:HGNC Symbol;Acc:HGNC:12524] |
| *PLP2* | proteolipid protein 2 [Source:HGNC Symbol;Acc:HGNC:9087] |
| *IL6ST* | interleukin 6 signal transducer [Source:HGNC Symbol;Acc:HGNC:6021] |
| *KCTD12* | potassium channel tetramerization domain containing 12 [Source:HGNC Symbol;Acc:HGNC:14678] |
| *TMEM123* | transmembrane protein 123 [Source:HGNC Symbol;Acc:HGNC:30138] |
| *PEA15* | phosphoprotein enriched in astrocytes 15 [Source:HGNC Symbol;Acc:HGNC:8822] |
| *ETS2* | ETS proto-oncogene 2, transcription factor [Source:HGNC Symbol;Acc:HGNC:3489] |
| *CAP1* | adenylate cyclase associated protein 1 [Source:HGNC Symbol;Acc:HGNC:20040] |
| *GARS* | glycyl-tRNA synthetase [Source:HGNC Symbol;Acc:HGNC:4162] |
| *JUND* | JunD proto-oncogene, AP-1 transcription factor subunit [Source:HGNC Symbol;Acc:HGNC:6206] |
| *NAMPT* | nicotinamide phosphoribosyltransferase [Source:HGNC Symbol;Acc:HGNC:30092] |
| *PTP4A2* | protein tyrosine phosphatase type IVA, member 2 [Source:HGNC Symbol;Acc:HGNC:9635] |
| *ACSL1* | acyl-CoA synthetase long-chain family member 1 [Source:HGNC Symbol;Acc:HGNC:3569] |
| *EWSR1* | EWS RNA binding protein 1 [Source:HGNC Symbol;Acc:HGNC:3508] |
| *DDX18* | DEAD-box helicase 18 [Source:HGNC Symbol;Acc:HGNC:2741] |
| *UFD1L* | ubiquitin fusion degradation 1 like (yeast) [Source:HGNC Symbol;Acc:HGNC:12520] |
| *ANXA1* | annexin A1 [Source:HGNC Symbol;Acc:HGNC:533] |
| *CAV1* | caveolin 1 [Source:HGNC Symbol;Acc:HGNC:1527] |
| *ACTN1* | actinin alpha 1 [Source:HGNC Symbol;Acc:HGNC:163] |
| *UAP1* | UDP-N-acetylglucosamine pyrophosphorylase 1 [Source:HGNC Symbol;Acc:HGNC:12457] |
| *FEN1* | flap structure-specific endonuclease 1 [Source:HGNC Symbol;Acc:HGNC:3650] |
| *IL1R1* | interleukin 1 receptor type 1 [Source:HGNC Symbol;Acc:HGNC:5993] |
| *CD59* | CD59 molecule [Source:HGNC Symbol;Acc:HGNC:1689] |
| *TIMP2* | TIMP metallopeptidase inhibitor 2 [Source:HGNC Symbol;Acc:HGNC:11821] |
| *IDH2* | isocitrate dehydrogenase (NADP(+)) 2, mitochondrial [Source:HGNC Symbol;Acc:HGNC:5383] |
| *ARF4* | ADP ribosylation factor 4 [Source:HGNC Symbol;Acc:HGNC:655] |
| *TUBB2A* | tubulin beta 2A class IIa [Source:HGNC Symbol;Acc:HGNC:12412] |
| *RRM2* | ribonucleotide reductase regulatory subunit M2 [Source:HGNC Symbol;Acc:HGNC:10452] |
| *RAN* | RAN, member RAS oncogene family [Source:HGNC Symbol;Acc:HGNC:9846] |
| *ID2* | inhibitor of DNA binding 2, HLH protein [Source:HGNC Symbol;Acc:HGNC:5361] |
| *SSRP1* | structure specific recognition protein 1 [Source:HGNC Symbol;Acc:HGNC:11327] |
| *ANXA4* | annexin A4 [Source:HGNC Symbol;Acc:HGNC:542] |
| *WARS* | tryptophanyl-tRNA synthetase [Source:HGNC Symbol;Acc:HGNC:12729] |
| *BHLHE40* | basic helix-loop-helix family member e40 [Source:HGNC Symbol;Acc:HGNC:1046] |
| *FERMT2* | fermitin family member 2 [Source:HGNC Symbol;Acc:HGNC:15767] |
| *TXNIP* | thioredoxin interacting protein [Source:HGNC Symbol;Acc:HGNC:16952] |
| *IFITM1* | interferon induced transmembrane protein 1 [Source:HGNC Symbol;Acc:HGNC:5412] |
| *TXN* | thioredoxin [Source:HGNC Symbol;Acc:HGNC:12435] |
| *GADD45A* | growth arrest and DNA damage inducible alpha [Source:HGNC Symbol;Acc:HGNC:4095] |
| *B2M* | beta-2-microglobulin [Source:HGNC Symbol;Acc:HGNC:914] |
| *LDHA* | lactate dehydrogenase A [Source:HGNC Symbol;Acc:HGNC:6535] |
| *STAT1* | signal transducer and activator of transcription 1 [Source:HGNC Symbol;Acc:HGNC:11362] |
| *CD55* | CD55 molecule (Cromer blood group) [Source:HGNC Symbol;Acc:HGNC:2665] |
| *ADD3* | adducin 3 [Source:HGNC Symbol;Acc:HGNC:245] |
| *DPYSL2* | dihydropyrimidinase like 2 [Source:HGNC Symbol;Acc:HGNC:3014] |
| *GLRX* | glutaredoxin [Source:HGNC Symbol;Acc:HGNC:4330] |
| *ITGA6* | integrin subunit alpha 6 [Source:HGNC Symbol;Acc:HGNC:6142] |
| *DUSP5* | dual specificity phosphatase 5 [Source:HGNC Symbol;Acc:HGNC:3071] |
| *SCP2* | sterol carrier protein 2 [Source:HGNC Symbol;Acc:HGNC:10606] |
| *CTSL* | cathepsin L [Source:HGNC Symbol;Acc:HGNC:2537] |
| *GCH1* | GTP cyclohydrolase 1 [Source:HGNC Symbol;Acc:HGNC:4193] |
| *CEBPD* | CCAAT/enhancer binding protein delta [Source:HGNC Symbol;Acc:HGNC:1835] |
| *VIM* | vimentin [Source:HGNC Symbol;Acc:HGNC:12692] |
| *PNP* | purine nucleoside phosphorylase [Source:HGNC Symbol;Acc:HGNC:7892] |
| *IFNGR1* | interferon gamma receptor 1 [Source:HGNC Symbol;Acc:HGNC:5439] |
| *TIMP1* | TIMP metallopeptidase inhibitor 1 [Source:HGNC Symbol;Acc:HGNC:11820] |
| *CDK1* | cyclin dependent kinase 1 [Source:HGNC Symbol;Acc:HGNC:1722] |
| *HNRNPAB* | heterogeneous nuclear ribonucleoprotein A/B [Source:HGNC Symbol;Acc:HGNC:5034] |
| *TUBG1* | tubulin gamma 1 [Source:HGNC Symbol;Acc:HGNC:12417] |
| *TUBA1A* | tubulin alpha 1a [Source:HGNC Symbol;Acc:HGNC:20766] |
| *MCM3* | minichromosome maintenance complex component 3 [Source:HGNC Symbol;Acc:HGNC:6945] |
| *CCNA2* | cyclin A2 [Source:HGNC Symbol;Acc:HGNC:1578] |
| *EEF2* | eukaryotic translation elongation factor 2 [Source:HGNC Symbol;Acc:HGNC:3214] |
| *PHB2* | prohibitin 2 [Source:HGNC Symbol;Acc:HGNC:30306] |
| *NPM1* | nucleophosmin (nucleolar phosphoprotein B23, numatrin) [Source:HGNC Symbol;Acc:HGNC:7910] |
| *FLNA* | filamin A [Source:HGNC Symbol;Acc:HGNC:3754] |
| *CDK4* | cyclin dependent kinase 4 [Source:HGNC Symbol;Acc:HGNC:1773] |
| *IFI16* | interferon gamma inducible protein 16 [Source:HGNC Symbol;Acc:HGNC:5395] |
| *MYC* | v-myc avian myelocytomatosis viral oncogene homolog [Source:HGNC Symbol;Acc:HGNC:7553] |
| *YWHAZ* | tyrosine 3-monooxygenase/tryptophan 5-monooxygenase activation protein zeta [Source:HGNC Symbol;Acc:HGNC:12855] |
| *HNRNPA1* | heterogeneous nuclear ribonucleoprotein A1 [Source:HGNC Symbol;Acc:HGNC:5031] |
| *ZFP36* | ZFP36 ring finger protein [Source:HGNC Symbol;Acc:HGNC:12862] |
| *JUNB* | JunB proto-oncogene, AP-1 transcription factor subunit [Source:HGNC Symbol;Acc:HGNC:6205] |
| *NFKBIA* | NFKB inhibitor alpha [Source:HGNC Symbol;Acc:HGNC:7797] |
| *FOS* | Fos proto-oncogene, AP-1 transcription factor subunit [Source:HGNC Symbol;Acc:HGNC:3796] |
| *ANXA5* | annexin A5 [Source:HGNC Symbol;Acc:HGNC:543] |
| *CDKN1A* | cyclin dependent kinase inhibitor 1A [Source:HGNC Symbol;Acc:HGNC:1784] |
| *HIF1A* | hypoxia inducible factor 1 alpha subunit [Source:HGNC Symbol;Acc:HGNC:4910] |
| *MYH9* | myosin, heavy chain 9, non-muscle [Source:HGNC Symbol;Acc:HGNC:7579] |
| *S100A10* | S100 calcium binding protein A10 [Source:HGNC Symbol;Acc:HGNC:10487] |
| *CEBPB* | CCAAT/enhancer binding protein beta [Source:HGNC Symbol;Acc:HGNC:1834] |
| *IER3* | immediate early response 3 [Source:HGNC Symbol;Acc:HGNC:5392] |
| *CAPN2* | calpain 2 [Source:HGNC Symbol;Acc:HGNC:1479] |
| *KLF10* | Kruppel like factor 10 [Source:HGNC Symbol;Acc:HGNC:11810] |
| *PLSCR1* | phospholipid scramblase 1 [Source:HGNC Symbol;Acc:HGNC:9092] |
| *SLC2A3* | solute carrier family 2 member 3 [Source:HGNC Symbol;Acc:HGNC:11007] |
| *TNFAIP3* | TNF alpha induced protein 3 [Source:HGNC Symbol;Acc:HGNC:11896] |
| *LGALS1* | galectin 1 [Source:HGNC Symbol;Acc:HGNC:6561] |
| *DUSP6* | dual specificity phosphatase 6 [Source:HGNC Symbol;Acc:HGNC:3072] |
| *SDCBP* | syndecan binding protein [Source:HGNC Symbol;Acc:HGNC:10662] |
| *CYR61* | cysteine rich angiogenic inducer 61 [Source:HGNC Symbol;Acc:HGNC:2654] |
